# Fungicide Resistance Evolving in *Ramularia collo-cygni* Population in Estonia

**DOI:** 10.3390/microorganisms9071514

**Published:** 2021-07-15

**Authors:** Riinu Kiiker, Marite Juurik, Andres Mäe

**Affiliations:** Department of Plant Protection, Estonian Crop Research Institute, 48309 Jõgeva, Estonia; riinu.kiiker@etki.ee (R.K.); marite.juurik@etki.ee (M.J.)

**Keywords:** Ramularia leaf spot, fungicide target proteins, CYP51, CytB, azoles, SDHI, QoI

## Abstract

Ramularia leaf spot caused by the fungus *Ramularia collo-cygni*, has recently become widespread in Estonian barley fields. Currently, disease control in barley fields relies on SDHI and DMI fungicides, which might be threatened by *R. collo-cygni* isolates that are well-adapted to fungicide pressure. In a two-year study, 353 *R. collo-cygni* isolates were collected from spring barley fields in Estonia. A total of 153 *R. collo-cygni* isolates were examined for sensitivity to azoles (DMIs; prothioconazole-desthio, epoxiconazole, mefentrifluconazole) and succinate dehydrogenase inhibitors (SDHIs; boscalid, fluxapyroxad). Epoxiconazole was the least effective and a new fungicide mefentrifluconazole was the most effective DMI. Among SDHIs, fluxapyroxad was more effective than boscalid. Also, single *R. collo-cygni* isolates with high resistance to tested fungicides occurred, which could affect fungicide control of the pathogen. The entire collection of *R. collo-cygni* was analysed for mutations in fungicide target proteins. Six mutations were identified in *CYP51* gene, the most dominant being I381T, I384T, and S459C. Also, numerous point mutations in the *SdhC* gene were present. The mutation G143A in strobilurin target protein CytB dominates in over 80% of the *R. collo-cygni* population, confirming the low efficacy of strobilurin fungicides in barley disease control.

## 1. Introduction

Barley (*Hordeum vulgare* L.) is the fourth most relevant cereal crop, and is grown in diverse environments worldwide [1]. Growing conditions in Estonia are well-suited for both spring and winter barley, and barley is the second most cultivated crop after wheat [2]. Barley yields are threatened by various pests and diseases [3]. Ramularia leaf spot (RLS) caused by *Ramularia collo-cygni* is a late-season disease that has emerged as a major problem in barley production in Europe, South America, and New Zealand over the last 20 years [3,4,5]. The first case of RLS was identified in Estonia 9 years ago in 2012 in a spring barley field [6], but in recent years RLS has become more widespread and is found every season in the majority of the spring and winter barley fields in Estonia.

The pathogen, the Dothideomycete fungus *R. collo*-*cygni* [7] can disperse by air-borne asexual spores and sexual ascospores during the growing season and via global seed transport between countries [8,9]. The pathogen infects barley seeds, where it is adapted to survive, and during plant development it colonises the emerging leaf layers in the absence of external inoculum [9]. *R. collo-cygni* grows initially as an endophyte and after a long latent period, it can then undergo a developmental switch to become an aggressive necrotrophic pathogen, initiated by several factors (e.g., nutrient deficiency, plant senescence) [10,11,12]. In diseased plants, RLS symptoms typically develop after ear emergence, although symptoms can be observed earlier in the season in especially conducive environmental conditions [5,13]. Expression of diverse *R. collo-cygni* virulence factors at the time of leaf infection is likely to promote RLS disease symptoms [14].

*R. collo-cygni* populations across Europe from the UK to Poland are highly adaptive because of high diversity within populations and novel genotypes are found every season [15,16]. Barley fields are threatened by another widespread barley pathogen *Pyrenophora teres,* which is also a genotypically highly diverse population [17,18]. In light of these results, more effort is needed to breed sustainable barley cultivars with multiple sources of host-plant resistance. So far, cultivars with full resistance to RLS are not available. However, transgenic barley plants which overexpressed *Stressed-related NAC1* (*SNAC1*), encoding a transcription factor involved in drought tolerance in cereals, had delayed leaf senescence and higher resistance to RLS [19]. In contrast, the broad-spectrum barley powdery mildew resistance gene *mlo*, which regulates a spontaneous lesion mimic phenotype [20] and is associated with accelerated leaf senescence and host cell death [21], is a major genetic factor conferring susceptibility to RLS in barley [22].

The risk of RLS epidemics is predicted to rise as the global climate changes [23], but defining the factors triggering the transition of *R*. *collo*-*cygni* from being an endophyte to a necrotrophic pathogen needs further comprehensive studies. The analyses, combining both barley and *R. collo-cygni* transcript profiles, show the activation of complex transcriptional programs in both organisms. Disease development was related to gene expression patterns similar to those found at the beginning of leaf senescence, when nutrients are possibly used by the infecting fungus [14]. Exposure of spring barley cultivars and a grass species *Brachypodium distachyon* to abiotic stresses, such as high light intensity, results in enhanced RLS symptom development [24]. If abiotic stress factors are required to elicit the fungal transition from endophyte to a pathogen, crops with enhanced tolerance to abiotic stress may also be more resistant to RLS.

In Estonia, net blotch (*Pyrenophora teres*) and spot blotch (*Cohliobolus sativus*) have been the most common foliar diseases of barley, causing serious yield and quality reduction [25]. Due to the increasing spread of *R. collo-cygni*, RLS has become an emerging disease in Estonian barley fields in the recent past [26]. Genetic studies have revealed a lack of geographical clustering of *R. collo-cygni* populations, which emphasises the major role of global seed transport in transmitting the pathogen around barley growing countries and also spreading fungicide resistant *R. collo-cygni* isolates [16,27].

There is a substantial risk of *R. collo*-*cygni* developing resistance to fungicides according to the Fungicide Resistance Action Committee (FRAC) [28]. *R. collo*-*cygni* has already acquired resistance to quinone-outside inhibitors (QoIs), a single-site inhibiting fungicide class that had high efficacy in the past [15,29,30]. For several years, disease control has been accomplished by two single-site fungicide classes, the succinate dehydrogenase inhibitors (SDHIs) and the demethylation inhibitors (DMIs), and the multi-site inhibitor, chlorothalonil [4]. In spite of the high efficacy of chlorothalonil against RLS [31], the European Food Safety Association (EFSA) banned the use of chlorothalonil-based fungicide products from 2020 onwards due to environmental safety concerns and high risk to amphibians and fish [32]. Consequently, effective management of RLS has to rely more on alternative measures and an integrated approach.

Resistance to SDHIs in the field has so far rarely been detected in the relevant cereal pathogens. Several mutations (e.g., B-H266Y/R, B-T267I, B-I268V, C-N87S, C-H146R, C-H153R) occur more frequently in the target genes of SDHIs in the population of *R. collo*-*cygni* [33]. Moreover, previous studies to create laboratory mutants of *R. collo*-*cygni* insensitive to SDHIs [34], as well as the detection of the first SDHI insensitive *R. collo*-*cygni* field isolates in Germany in 2014 and 2015 [35], have led to concerns about the field performance of SDHIs in the future. Recently, amino acid alterations C-H146R and C-H153R in SDH subunits, which confer a noticeable decrease in sensitivity in bioassays, and C-N87S with a lower reduction in sensitivity, were detected in *R. collo-cygni* isolates from different European regions [33,35]. Recent examples of reduced SDHI sensitivity in other cereal pathogens *P. teres* [36] and *Zymoseptoria tritici* [37,38] highlight the risk of SDHI resistance evolution.

DMIs have been applied for the control of cereal diseases for more than three decades, which has led to the emergence of adapted strains of cereal pathogens [39,40]. Mutations in the coding region of *CYP51* are the most eminent mechanism for DMI adaptation in many fungal pathogens [41,42]. In total, 12 alterations and 15 different *R. collo-cygni CYP51* haplotypes were identified in 2017 in Europe [33]. The most frequent haplotype was the *C1* with mutations I381T, I384L, and Y459C in *CYP51* and the second most popular haplotype C3 had a combination of I381T, I384L, and Y461H mutations [33]. In contrast to this, *CYP51* evolution and other resistance mechanisms (e.g., the combination of target site alterations with promoter insertions or an enhanced efflux) have not been studied so far in *R. collo*-*cygni*.

Since the beginning of 2000, RLS has been identified in crops and seed samples from the Scandinavian and Baltic countries, for instance, Finland, Sweden (2001), Denmark (2002), Lithuania (2004) Latvia (2011), and Estonia (2012) [4,6,43]. It is difficult to predict the capacity of pathogen populations because RLS is very often hidden behind other diseases and the diagnosis of physiological leaf spots [44]. In 2012, *R. collo-cygni* was first found in a few spring barley fields in Estonia [6], but in the growing season of 2015–2016, RLS infection was more widespread and had also spread to winter barley fields [26].

Since there is a high probability of spread and the evolvement of fungicide resistance in *R. collo-cygni* populations, protecting the crop efficiently with fungicides becomes even more critical. The current study was prompted by reports of the continuous spread of *R. collo-cygni* in barley fields in Estonia. Therefore, *R. collo-cygni* isolates were collected from commercial spring barley fields in 2019 and 2020. The first objective of this study was to explore the development of molecular resistance in fungicide target genes in *R. collo-cygni* isolates. Our second objective was to analyze the development of fungicide resistance to different DMI (epoxiconazole, prothioconazole-desthio, and mefentrifluconazole) and SDHI (fluxapyroxad and boscalid) fungicides applied in barley disease control.

## 2. Materials and Methods

### 2.1. Isolate Collection

**Figure 1:**
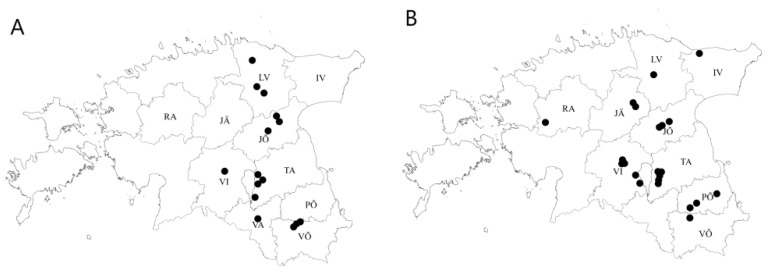
Spring barley fields of *R. collo-cygni* collection in 2019 (**A**) and 2020 (**B**) in Estonia.

In the growing seasons of 2019 and 2020, leaf samples with naturally occurring *R. collo-cygni* infection were gathered from fungicide-treated commercial fields of spring barley (BBCH 83–89) across Estonia (Figure 1). Samples originated from six (Jõgeva, Lääne-Viru, Viljandi, Tartu Võru and Valga) and nine (Ida-Viru, Lääne-Viru, Järva, Jõgeva, Põlva, Rapla, Tartu, Viljandi and Võru) counties in 2019 and 2020, respectively (Figure 1; Table 1). RLS chemical control varied from one to two fungicide applications (with products containing mainly prothioconazole, epoxiconazole, boscalid, fluxapyroxad, bixafen, and pyraclostrobin) up to the label dose in these fields. In the commercial fields, the average field concentrations of fungicides for active ingredients analysed in the current study were 67 g a.i/ha for epoxiconazole, 75 g a.i/ha for fluxapyroxad, 140 g a.i/ha for boscalid, and 180 g a.i/ha for prothioconazole.

Single-spore cultures of *R. collo-cygni* were isolated directly from barley leaves using a fine sterile insect pin. All the *R. collo-cygni* isolates were grown on potato dextrose agar (PDA) medium supplemented with streptomycin 5 μg mL^−1^ in the dark at 17 °C and maintained at 12 °C until further analysis.

### 2.2. Determination of Fungicide Sensitivity

*R. collo-cygni* isolates were cultivated in 5 mL of potato dextrose broth (PDB) in a 15 mL Falcon tube before fungicide sensitivity analysis. For that, 0.5 cm^2^ section of the agar block was cut from the edge of the *R. collo-cygni* isolate on PDA plate, and cultured in 5 mL of PDB for 10–12 days in the dark at 17 °C, shaking at 120 rpm. Subsequently, 5 mL of each culture was homogenised for 2 min at 24,000 rpm using TissueRuptor^®^ II homogeniser (Qiagen, GmbH, Germany) with sterilised reusable plastic blades (Qiagen, GmbH, Germany). The suspensions were vortex-mixed in 15 mL Falcon tubes for 15 min for homogenisation at 2000 rpm using the Multi Reax vortexer (Heidolph Instruments GmbH & CO, Schwabach, Germany). Each culture was diluted until the final concentration of 2.5 × 10^3^ fragments of mycelium per mL.

In 2019, 38 *R. collo-cygni* isolates and in 2020, 115 isolates were analysed for fungicide sensitivity for five fungicide active ingredients in a microtiter plate assay (Table 1). Epoxiconazole, prothioconazole-desthio, boscalid, fluxapyroxad (all Sigma-Aldrich, St. Louis, MO, USA), and mefentrifluconazole (LGC Dr. Ehrenstorfer, Augsburg, Germany) were diluted separately with 2 × PDB to gain the following final microtiter plate fungicide concentrations (ppm): 30, 6, 1.2, 0.24, 0.048, 0.01, 0.002, and 0 for epoxiconazole; 6, 2, 0.67, 0.22, 0.074, 0.025, 0.008, and 0 for prothioconazole-desthio; 3, 1, 0.33, 0.11, 0.037, 0.012, 0.004, and 0 for mefentrifluconazole and fluxapyroxad; 10, 3.3, 1.1, 0.37, 0.12, 0.041, 0.014 and 0 for boscalid. Mycelium suspension and fungicide dilutions were added to a nuncTM 96-deep well microtiter plate (Thermo Fisher Scientific, Roskilde, Denmark) in equal amounts of 100 µL. Each isolate was duplicated on the same plate, and Dutch isolate DK05 (azole-sensitive) was included as a reference in each assay. Microtiter plates were sealed and covered with aluminum foil and incubated in the dark for six days at 17 °C with shaking at 120 rpm. The plates were visually assessed for biological contamination before measuring the optical density in a Tecan Sunrise Microplate Absorbance Reader (Tecan, Männedorf, Switzerland) at a wavelength 620 nm. The half-maximal effective concentration (EC_50_) of each fungicide was determined by non-linear regression (curve-fit) using GraphPad Prism version 9.0.2 (GraphPad Software, La Jolla, CA, USA). Resistance factors (RF) were determined by the formula: RF = (mean EC_50_ of *R. collo-cygni* population)/(mean EC_50_ of reference isolate DK05).

### 2.3. Identifying Target Site Mutations in the Genes of Fungicide Target Proteins

Genomic DNA from *R. collo-cygni* isolates was extracted by the thermolysis method [45]. All the PCR reactions were done in a 25 µL volume consisting of 10.9 µL MilliQ water, 5.0 µL 5 × DreamTaq PCR buffer (Thermo Fisher Scientific, Waltham, MA, USA), 100 µM of each dNTP, 10 µM of specific forward and reverse primers (Table S1), 1 unit of DreamTaq DNA polymerase (Thermo Fisher Scientific, Waltham, MA, USA), and 1.0 µL DNA (about 10 ng µL^−1^).

PCR reactions to obtain the sequences of the *Sdh* gene were performed using primers SdhB_Rcc_Final_F and SdhB_Rcc_Final_R for SdhB subunit and SdhC_Rcc_Final_F and SdhC_Rcc_Final_R for SdhC subunit (Table S1) [34]. The amplification was performed using the following conditions: initial denaturation at 95 °C for 2 min, followed by 30 cycles of denaturation at 95 °C for 15 s, annealing at 57 °C (SdhB) or 50 °C (SdhC) for 30 s, extension at 72 °C for 1 min and a final extension at 72 °C for 10 min.

The amplification of the *CYP51* gene was performed using primers KES2230 and KES2231 for amplification (Table S1) [33]. The conditions for amplification were: initial denaturation at 95 °C for 60 s, 35 cycles at 95 °C for 30 s, 60 °C for 30 s, 72 °C for 120 s and a final elongation step at 72 °C for 5 min.

The amplification of the *CytB* gene was performed using primers RCCcytobF and RCCcytobR for amplification (Table S1) [30]. The conditions for amplification were: initial denaturation at 95 °C for 90 s, 35 cycles at 95 °C for 60 s, 55 °C for 45 s, 72 °C for 120 s and a final elongation step at 72 °C for 5 min.

All PCR products were sequenced using the same forward and reverse primers using an Applied Biosystems 3730 DNA Analyzer (Thermo Fisher Scientific, Waltham, MA, USA) in the Estonian Biocentre in Tartu. The sequences obtained were analysed against non-redundant databases and DK05 reference sequence using blastn and blastx search tools publicly available at NCBI [46]. Target-site mutations in each gene were identified.

### 2.4. Statistical Analysis

SuperPlotsOfData Shiny app was applied for visualising the EC_50_ results [47]. For statistical analyses, GraphPad Prism (GraphPad Software, La Jolla, CA, USA) was implemented. An unpaired t-test with Welch’s correction was conducted to compare the mean EC_50_ values of each fungicide from the *R. collo-cygni* collection from two study years (α = 0.05). The Kruskal–Wallis test with Dunn’s multiple comparison test was performed to compare EC_50_ values between counties (α = 0.05). Pearson correlation analysis for log-transformed EC_50_ values for pairs of azole fungicides was performed.

## 3. Results

To study the fungicide sensitivity of the Estonian *R. collo*-*cygni* population, a total of 353 isolates were collected from spring barley plants in the ripening stage before harvest (in July, August) in 2019 and 2020 (Table 1). The samples were collected from commercial barley fields, where crop protection fungicides had been applied. The *R. collo*-*cygni* population was tested for active ingredients of DMI (epoxiconazole, prothioconazole-desthio, and mefentrifluconazole) and SDHI (boscalid and fluxapyroxad) class fungicides, and also relevant mutations in fungicide target genes (*CYP51*, *SdhC*, and *CytB*) were identified.

### 3.1. Status of DMI Fungicide Sensitivity

**Figure 2:**
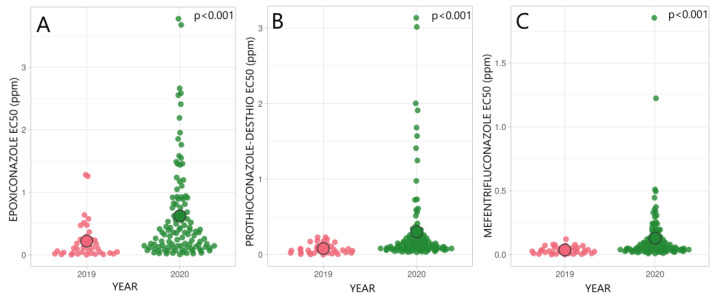
Sensitivity of *R. collo-cygni* population in 2019 and 2020 in Estonia to DMI fungicides epoxiconazole (**A**), prothioconazole-desthio (**B**), and mefentrifluconazole (**C**). ○ indicates average EC_50_ value (ppm) in a population; *p* < 0.001 shows a significant difference between the years according to the unpaired *t*-test with Welch’s correction.

In 2019, 38 *R. collo-cygni* isolates from five Estonian counties, and in 2020, 115 isolates from nine counties were analysed in fungicide sensitivity microtiter plate assay. In the *R. collo-cygni* population, there was a significant increase in EC_50_ values of epoxiconazole (t_143_ = 4.69; *p* < 0.001), prothioconazole-desthio (t_124_ = 4.43; *p* < 0.001), and mefentrifluconazole (t_121_ = 4.32; *p* < 0.001) in 2020 compared to 2019 (Figure 2). In this period the average EC_50_ values for epoxiconazole varied from 0.22 ppm in 2019 to 0.63 ppm in 2020. In the same period, prothioconazole-desthio EC_50_ average values increased from 0.08 to 0.3 ppm. Sensitivity towards mefentrifluconazole remained high with average EC_50_ values ranging from 0.04 to 0.13 ppm. In the Estonian *R. collo-cygni* population, a considerable proportion of isolates with higher EC_50_ values (>1.0 ppm) for epoxiconazole and prothioconazole-desthio were found in 2020 compared to 2019.

The EC_50_ values were further analysed with the Pearson correlation, which confirmed high positive correlation between prothioconazole-desthio and mefentrifluconazole sensitivity (r = 0.574, *p* < 0.001). A significant correlation was also detected between epoxiconazole and prothioconazole-desthio (r = 0.243, *p* = 0.003) and epoxiconazole and mefentrifluconazole sensitivity (r = 0.236, *p* = 0.004).

**Figure 3:**
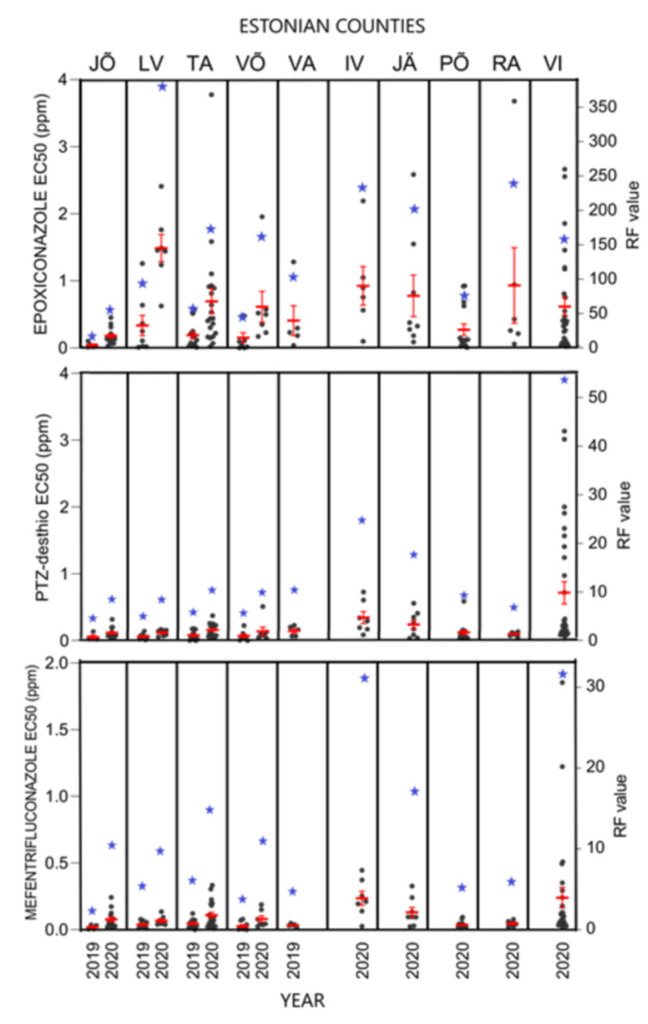
DMI fungicide sensitivity in *R. collo-cygni* population in Estonia by counties and collection years. Black dots show individual EC_50_ values (ppm), red error bars represent standard error of the mean EC_50_ values, and blue stars are average resistance factor (RF) values. Counties presented are: JÕ—Jõgeva; LV—Lääne-Viru; TA—Tartu; VÕ—Võru; VA—Valga; IV—Ida-Viru; JÄ—Järva; PÕ—Põlva; RA—Rapla; VI—Viljandi.

Figure 3 visualises the individual and average EC_50_ values and resistance factors (RF) for epoxiconazole, prothioconazole-desthio, and mefentrifluconazole in different counties from 2019 to 2020. There was a significant difference between the counties among all tested fungicides; for epoxiconazole (KW-H = 22.22, *p* = 0.008), prothioconazole-desthio (KW-H = 40.79, *p* < 0.001), and mefentrifluconazole (KW-H = 33.17, *p* < 0.001). From 2019 to 2020, sensitivity towards epoxiconazole decreased in Lääne-Viru with average EC_50_ values of 0.33 ppm and 1.49 ppm (RF = 87–389), Tartu with average EC_50_ values of 0.19 and 0.70 ppm (RF = 49–181), and Võru counties with average EC_50_ values of 0.15 and 0.61 ppm (RF = 40–160), respectively. Sensitivity towards epoxiconazole remained more stable between 2019 and 2020 in Jõgeva with average EC_50_ values ranging from 0.04 to 0.18 ppm (RF = 11–47), respectively. In Valga, the average EC_50_ was 0.41 ppm (RF = 106) in 2019. From Ida-Viru, Järva, Põlva, Rapla, and Viljandi counties we had *R. collo-cygni* isolates only for 2020; the average EC_50_ values between these counties varied from 0.27 to 0.93 ppm (RF = 71–243) (Figure 3).

In the same period, sensitivity towards prothioconazole-desthio was higher compared to epoxiconazole in *R. collo-cygni* population. A relatively high EC_50_ value for prothioconazole-desthio was seen in Viljandi county with an average value of 0.72 ppm (RF = 55) in 2020. In other counties, sensitivity towards prothioconazole-desthio was lower in both study years, with average EC_50_ values ranging from 0.05 to 0.35 ppm (RF = 4–27) (Figure 3).

The *R. collo-cygni* population was the most sensitive to a new azole fungicide mefentrifluconazole with average EC_50_ values ranging from 0.02 to 0.24 ppm (RF = 3–32). Higher values were observed in Ida-Viru and Viljandi counties (Figure 3).

### 3.2. Mutations in CYP51 Gene

To identify the mutations in the *CYP51* gene that cause a change in adaptation level towards DMI fungicides, the whole *CYP51* gene in 68 (in 2019) and 285 (in 2020) *R. collo-cygni* isolates was sequenced (Table 1). Sequencing revealed six mutations in three positions, leading to alterations at amino acids (I381T, I384T, S459C/Y/T/L) in the CYP51 enzyme (Table 2). The level of *CYP51* mutations I381T, I384T, and S459C showed some variation between 2019 and 2020. The exceptions were mutations S459T and S459L, which disappeared from the population in 2020, and mutation S459Y, which only appeared in *R. collo-cygni* populations in 2020 (Table 2). In both years, the most frequent (93–96%) mutations I381T and I384T occurred together in the same *R. collo-cygni* isolates (Table 2). These mutations were present in 70–100% of the isolates across counties (Table 2). Single nucleotide mutations lead to amino acid changes from serine to tyrosine, cysteine, threonine, or leucine at position 459 (S459) in CYP51 enzyme. Mutation S459C was the most common, at an average frequency of 80% in 2019 and 79% in 2020. In 2020, a new mutation, S459Y, appeared with an average frequency of 16% in a population, which was present only in four counties, but at unexpectedly high frequencies in Jõgeva (54%) and Järva (63%) (Table 2). The mutation S459T was found only in two counties in 2019 and S459L was found only in Võru county at an average frequency of 17% in 2019 (Table 2).

### 3.3. Status of SDHI Fungicide Sensitivity

**Figure 4:**
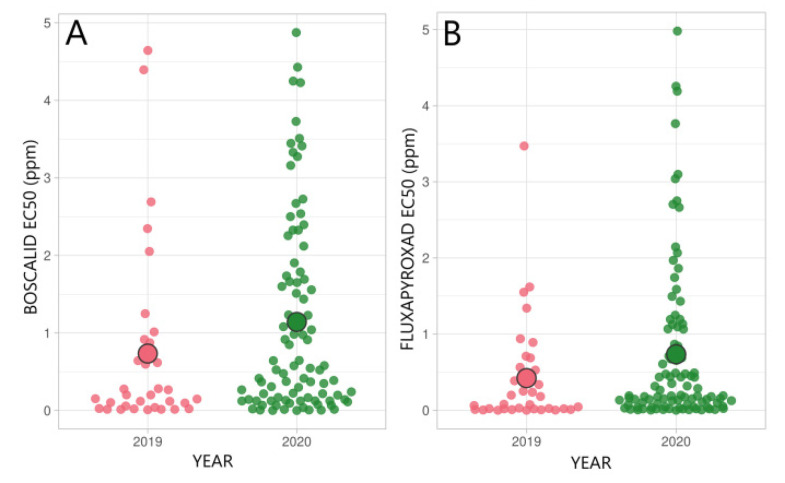
Sensitivity of *R. collo-cygni* in 2019 and 2020 in Estonia to SDHI fungicides boscalid (**A**) and fluxapyroxad (**B**). ○ shows average EC_50_ value (ppm) in a population.

Fluxapyroxad and boscalid were used for SDHI sensitivity analyses. Sensitivity to both fungicides was stable in the *R. collo-cygni* population between 2019 and 2020, although EC_50_ values showed a slightly moderate increase (Figure 4). Average EC_50_ values in 2019 and 2020 were 0.73 ppm (RF = 13) and 1.14 ppm (RF = 20) for boscalid and 0.42 ppm (RF = 17) and 0.74 ppm (RF = 29) for fluxapyroxad, respectively.

**Figure 5:**
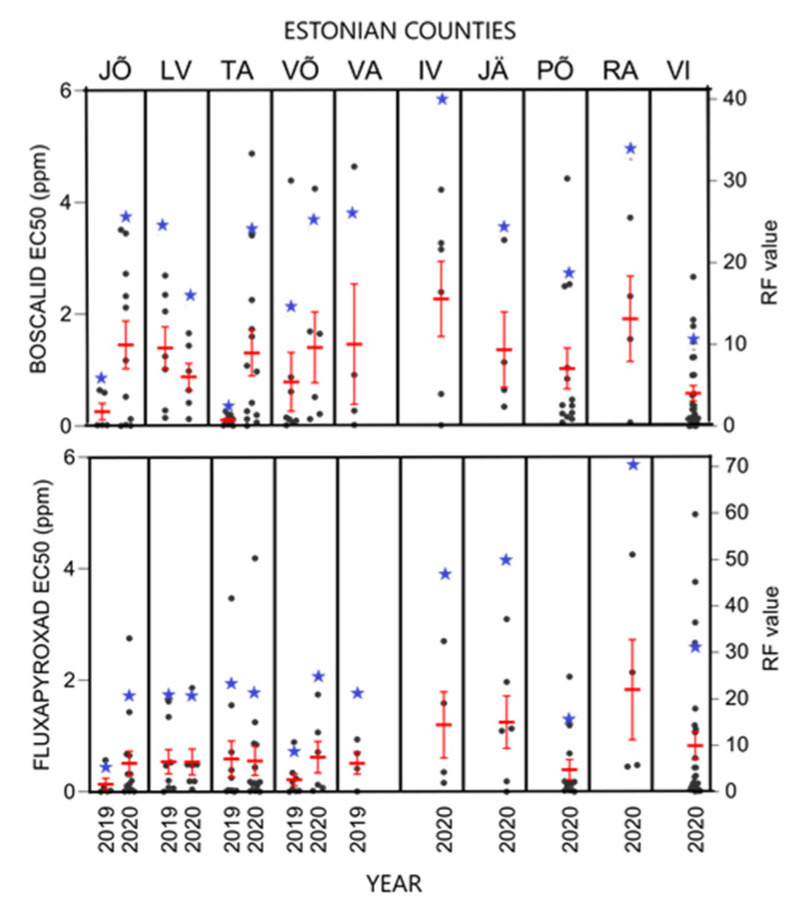
SDHI fungicide sensitivity in *R. collo-cygni* population in Estonia by counties and collection years. Black dots show individual EC_50_ values (ppm), red error bars represent standard error of the mean EC_50_ value, and blue stars are average resistance factor (RF) values. Counties presented are: JÕ—Jõgeva; LV—Lääne-Viru; TA—Tartu; VÕ—Võru; VA—Valga; IV—Ida-Viru; JÄ—Järva; PÕ—Põlva; RA—Rapla; VI—Viljandi.

EC_50_ values for boscalid and fluxapyroxad varied between different counties, but without significant differences (*p* > 0.05). EC_50_ varied between the counties from 0.26 ppm (RF = 5) to 1.46 ppm (RF = 26) in 2019 and from 0.59 (RF = 10) to 2.28 ppm (RF = 40) in 2020 for boscalid (Figure 5). In the same period, fluxapyroxad EC_50_ values in different counties varied between 0.13 ppm (RF = 5) and 0.59 ppm (RF = 23) in 2019 and between 0.41 ppm (RF = 16) and 1.83 ppm (RF = 72) in 2020.

### 3.4. Mutations in Sdh Protein Subunits

Several mutations were identified in the SdhC subunit, but none occurred in the SdhB subunit in the Estonian *R. collo-cygni* population. The most prevalent mutation C-H146R in the SdhC subunit showed a decreasing trend of average frequency from 63 to 55% from 2019 to 2020 (Table 3).

The frequency of C-H146R was highly variable between counties in both years. In 2019, the frequency of C-H146R varied from 40% in Valga county to 88% in Lääne-Viru county (Table 3). In 2020, the frequency of this mutation varied even from 0–100% between counties. This is the first identification of a C-V184L mutation in the SdhC subunit, which was found in 2019 in 11% of the isolates from Jõgeva county, but in 2020 this mutation had spread and occurred at frequencies between 20 and 50% in five counties (Table 3). Interestingly, in 2020 two new mutations C-N164H (28%) and C-G167C (29%) appeared in the Estonian *R. collo-cygni* population. Also, other mutations in the SdhC subunit (T7S, Q8R/P, Q9L, A65P, A81P, N83S, A121G, H153R, K156N, K161N, N164H/P, G167D, G171D) occurred at low frequency (1–15%).

### 3.5. Mutation G143A Prevalence in CytB Gene

**Figure 6:**
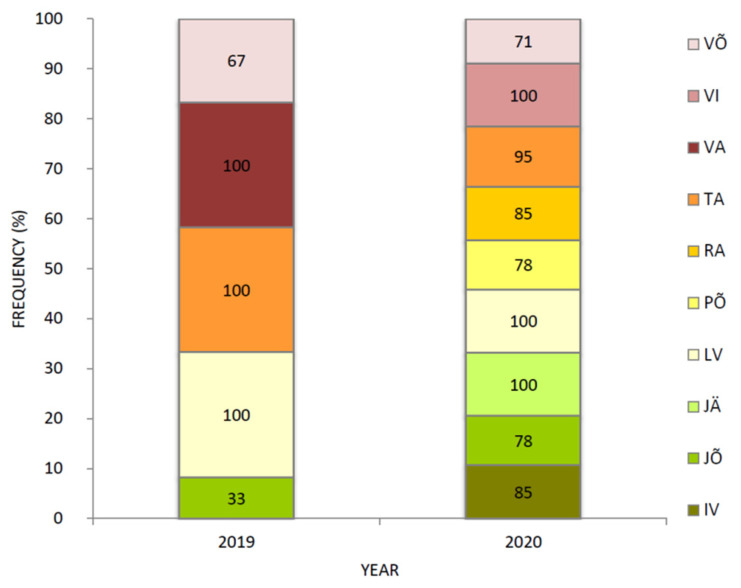
Mutation G143A frequency (%) in *CytB* gene in Estonian *R. collo-cygni* population within counties by collection years. Counties presented are: IV—Ida-Viru; JÕ—Jõgeva; JÄ—Järva; LV—Lääne-Viru; PÕ—Põlva; RA—Rapla; TA—Tartu; VA—Valga; VI—Viljandi; VÕ—Võru.

The mutation G143A in the cytochrome b (*CytB*) gene confirms resistance to QoI fungicides. Between 2019 and 2020, an increase occurred in the frequency of mutation G143A in *R. collo-cygni* population, from 80% in 2019 to 88% in 2020. The mutation G143A was present in all counties in both years (Figure 6). In 2020, the frequency had increased in Jõgeva county (from 33% to 78%) and Võru county (from 67% to 71%). In Järva, Lääne-Viru, Valga, and Viljandi county the mutation G143A was present in every *R. collo-cygni* isolate (Figure 6).

## 4. Discussion

The rapid evolvement of RLS disease during the past 10 years, together with the breakdown of fungicide efficacy led us to investigate the present fungicide resistance situation in the Estonian *R. collo-cygni* population. Currently, the chemical control of RLS relies on using synthetic products with different modes of action. A retrospective study was performed to evaluate the changes in fungicide sensitivity of *R. collo-cygni* population in commercial spring barley fields. The sensitivity of DMIs (epoxiconazole, prothioconazole-desthio, and mefentrifluconazole) and SDHIs (boscalid and fluxapyroxad) in the Estonian *R. collo-cygni* population has only been monitored since 2019. This approach was adopted to update the distribution of fungicide sensitivity to active ingredients commonly used in barley disease control.

RLS was first found in Estonia in 2012 in barley fields in Jõgeva county. Approximately 30% of the analysed plants were infected with RLS, with disease severity ranging from 30 to 50% between the fields [6]. During the period from 2012 to 2020, the disease spread throughout most of the barley growing areas in eastern and southern Estonia (Figure 1), affecting up to 90% of the barley fields monitored in 2020 around Estonia.

In this study, 353 *R. collo-cygni* isolates were collected in Estonia from ten different counties. We observed that the sensitivity to DMI and SDHI fungicides was highly variable in the *R. collo-cygni* population in Estonia during the years 2019–2020. The sensitivity towards epoxiconazole has decreased moderately from 2019 to 2020, with average EC_50_ values changing from 0.22 ppm to 0.63 ppm, respectively (Figure 2). The data presented in this paper showed that in comparison to 2019, an increase in the number of isolates with high EC_50_ values was found in 2020 (Figure 2). The appearance of less sensitive isolates in the population shows that these *R. collo*-*cygni* strains exist in the fields in different counties and should be considered a potential risk for the future. A similar trend for prothioconazole-desthio was observed with average EC_50_ values increasing from 0.05 ppm in 2019 to 0.30 ppm in 2020 (Figure 2). Interestingly, a high level of variability in sensitivity to prothioconazole-desthio (EC_50_ values from 0.07 to 3.14 ppm) and numerous isolates with EC_50_ values higher than 1, were found only in 2020 from Viljandi county (Figure 3).

Our data indicate that in the Estonian *R. collo-cygni* population fungicide resistance towards DMI class fungicides (epoxiconazole and prothioconazole-desthio) has developed more slowly in comparison to Western Europe. In Estonia, the climatic conditions are less conducive to RLS, and fungicides are therefore less intensively used. In regions with a high RLS distribution (e.g., UK, Germany, Austria), fungicides are used intensively, and since 2015, *R. collo-cygni* populations have significantly lost sensitivity to DMI fungicides. The first report came from Austria and a year later from UK and Germany [35]. As a result, DMIs are regarded as being ineffective against *R. collo-cygni* in these countries [48]. Intensive monitoring of barley fields by Syngenta demonstrated that DMI field efficacy against RLS decreased from nearly 100% in 2014 to around 30% in 2019 [49]. The reduction in the control of *R. collo*-*cygni* by DMIs has increased concerns for future management of RLS [35].

The sensitivity of a new promising fungicide a.i. mefentrifluconazole in the *R. collo-cygni* population has been monitored in Estonia since 2019. The first fungicide products Revytrex (a.i. fluxapyroxad and mefentrifluconazole; BASF Agro B.V.) and Balaya (a.i. pyraclostrobin and mefentrifluconazole; BASF Agro B.V.) have been on the Estonian market only for a year and applied on the commercial fields since 2021. This new azole class fungicide mefentrifluconazole could be used in RLS control as the efficacy was high in *R. collo-cygni* population, EC_50_ = 0.02 ppm in 2019; EC_50_ = 0.24 ppm in 2020 (Figure 2). Although mefentrifluconazole is a new active ingredient, sensitivity assay revealed a few, already less-sensitive isolates in the Estonian *R. collo-cygni* population in Viljandi county in 2020 (Figure 3). These findings might be explained by the differences in the regional use of fungicides, together with local agronomic and environmental factors. Thus, in cases where the population is still sensitive to mefentrifluconazole except for few single isolates, a mixture of or an alternation between mefentrifluconazole and other DMIs or SDHIs would be effective in RLS control. However, these approaches need careful evaluation and further research activities. In the current study, a high positive correlation between prothioconazole-desthio and mefentrifluconazole sensitivity was seen. It has also been shown that mefentrifluconazole and tebuconazole have cross-resistance patterns in *Z. tritici* isolates and care should be taken because of previous extensive applications of tebuconazole fungicide products in cereal plant protection [50,51].

Isolates with significant loss of sensitivity to DMI fungicides highlighted mutations in the target gene *CYP51* as the major resistance mechanism in *R. collo-cygni* [33,35]. A study across Europe carried out between 2009 and 2017, documented 12 *CYP51* alterations (V136A, Y137F, A311S, I381T, I384L, D458G, Y459C, Y459N, G460D, G460V, Y461N, and Y461H; based on *Z. tritici* *CYP51* sequence) and 15 different *CYP51* haplotypes [33]. The study pointed out that the haplotypes *C1* (mutations I381T, I384L, and Y459C) and *C3* (mutations I381T, I384L, and Y461H) were found in high frequencies and resulted in reduced field efficacy of DMI fungicides.

In our screening, the combination of I381T and I384T in *CYP51* was the most dominant with frequencies of 96% in 2019 and 93% in 2020. This agrees with findings from several European countries. Rehfus et al. [33] hypothesised that a single amino acid alteration in either position 381 or 384 is lethal and isolate can only survive when both mutations occur in the genome [33]. These two prevalent mutations were always combined with substitution in position S459 where serine was replaced either by C, Y, T, or L residue. Mutation S459C was the most common, at an average frequency of 80% in 2019 and 79% in 2020 (Table 2). In 2020, two mutations, S459L and S459T disappeared from the population while in 2020 a new mutation S459Y appeared (Table 2). In 2019 and 2020 the combination of I381T, I384T, and S459C/Y in *CYP51* became increasingly common in the Estonian *R. collo-cygni* population (Table 2). These combinations of mutations are very similar to *R. collo-cygni* haplotype *C1* and *C3*, referred to previously [33].

Single amino acid substitutions in SdhB, SdhC, and SdhD subunits have been shown to confer reduced sensitivity to SDHI fungicides. Mutations C-H146R and C-H153R, which influence the efficacy of SDHIs, show significant levels in Europe and have resulted in reduced RLS control, as described for France, Germany, UK, and Ireland by FRAC [35]. In the Estonian population, mutation C-H146R is widely distributed and reduced sensitivity to boscalid and fluxapyroxad also occurs in specific counties (Ida-Viru and Rapla) where the mutation frequency is high (Figure 5). Refhus et al. [33] analysed *R. collo-cygni* isolates collected from Western and Central Europe from 2014 to 2017 and revealed the lowest sensitivity to fluxapyroxad for isolates carrying alterations C-H146R/L, C-G171D, C-H153R, and C-G91R. However, the resistance profile of C-H142R (the same as C-H146R) is variable, being highly resistant to boscalid but moderately resistant to isopyrozame, bixafen, fluopyram, and carboxin [34]. Sequencing of Sdh subunits B (SdhB), and C (SdhC) revealed the presence of mutations only in subunit C in the Estonian *R. collo-cygni* population, the most frequent being C-H146R, C-N164H, C-G167C, and C-V184L. The three last-mentioned mutations were detected only in 2020, except for C-V184L, which was already found in Jõgeva county in 2019 (Table 3). The mutation C-H153R, which is strongly involved in the reduced efficacy of SDHIs, was found only in 2019 in low frequency (5%) in the Estonian *R. collo-cygni* population. The appearance of new mutations confirms the still ongoing evolution of fungicide resistance in the population. Although, in European *R. collo-cygni* samples, the first SDHI-resistant isolates were found in 2015 in Germany, Ireland, and Slovenia, and in 2017 in Austria [35], the principles of anti-resistance management should be applied to extend the effective life of SDHIs in the future.

In this study, sensitivity to SDHI fungicides was quite stable. Average EC_50_ values for boscalid slightly increased from 0.73 ppm (RF = 13) in 2019 to 1.14 ppm (RF = 20) in 2020, respectively. Simultaneously, average EC_50_ values for fluxapyroxad were 0.42 ppm (RF = 17) in 2019 and 0.74 ppm (RF = 29) in 2020 (Figure 4). In each county, except for Jõgeva, Tartu, and Võru, average RF values were between 10 and 100, which is considered moderately resistant [34]. Also, a high level of variability in sensitivities to SDHIs was found between samples from the same county. This is in line with Strobel et al. [44] who reported a wide range of EC_50_ values towards fluxapyoxad, with an obvious difference between the samples, even from the same field.

Quinone outside inhibitors (QoIs) were relatively effective at controlling RLS when it was first recognised as a potentially serious threat to barley crops in the late 1990s. The fungicide Amistar (azoxystrobin) was found to be very efficient for RLS disease control in most barley-growing regions. However, already during 2002, there was a sharp decline in the efficacy of azoxystrobin when used to control RLS [29]. The control of RLS by QoIs is hardly effective anymore against *R. collo-cygni* populations in the United Kingdom, Denmark, Czech Republic, and Germany [4,29,30].

The replacement of glycine (G) by alanine (A) at codon 143 in CytB is widely known to cause complete resistance to QoI fungicides and has been found in a range of plant pathogens, such as *Z. tritici* and *Mycosphaerella fijiensis,* which are closely related to *Ramularia* sp. [52]. For instance, in the Estonian *Z. tritici* population in recent years up to 50% of the isolates had G143A mutation in the *CytB* gene [51]. Our results showed high G143A mutation frequency and even a slight increase over the last two years in the *R. collo-cygni* population (80% in 2019 and 88% in 2020). Still, the frequency varied between different counties and study years. But the frequency of the QoI insensitive allele (G143A) exceeded 70% level in all counties (Figure 6). Even though not recommended anymore for RLS control, QoI fungicides remain effective against other diseases (e.g., rust diseases) and are still applied to protect the crop. The continuous use of strobilurins in commercial fields creates favorable conditions for the further spread of *R. collo-cygni* strains carrying G143A. In 2020, a member of a new generation of QoI metyltetraprole was successfully tested against QoI-resistant strains of pathogenic fungi with G143A mutation; all tested isolates were highly susceptible to metyltetraprole [53]. This new active ingredient is expected to replace some of the currently available QoIs due to its high intrinsic activity on *R. collo-cygni*, as shown in several laboratory and greenhouse tests [53].

## 5. Conclusions

RLS caused by the fungus *R. collo-cygni*, has recently become widespread in Estonia. The combination of long-distance dispersal, global seed transmission, and lack of resistant cultivars renders the control of RLS even more difficult. Currently, the control of disease in barley fields depends on SDHI and DMI fungicides. In general, fungicide sensitivity in the Estonian *R. collo-cygni* population is high or moderately declined. In our screening, we found single isolates showing high resistance to tested fungicides. This may contribute to wider dispersal of DMI and SDHI-resistance in a population, posing a challenge for further fungicide control of the pathogen during the next years. Also, numerous point mutations in the coding regions of *Sdh*, *CYP51,* and *CytB* genes contributing to the evolvement of fungicide resistance were found in the Estonian *R. collo-cygni* population. Future studies should seek to explain the sources of variation in local resistance evolution, identify factors determining resistance dynamics, and provide further guidelines for resistance management.

## Figures and Tables

**Table 1:** The number of *R. collo-cygni* collection fields and isolates analysed for mutations in fungicide target genes and fungicide sensitivity in microtiter plate assays by counties and years.

County *	Fields		Mutations		Fungicide Sensitivity Assay	
	2019	2020	2019	2020	2019	2020
IV	NA **	5	NA	64	NA	7
JÕ	2	3	9	35	5	15
JÄ	NA	7	NA	36	NA	8
LV	3	1	17	11	8	7
PÕ	NA	3	NA	15	NA	15
RA	NA	1	NA	6	NA	6
TA	4	6	20	63	12	20
VA	1	NA	5	NA	5	NA
VI	1	5	5	48	NA	30
VÕ	3	1	12	7	8	7
TOTAL	14	32	68	285	38	115

* Counties presented are: IV—Ida-Viru; JÕ—Jõgeva; JÄ—Järva; LV—Lääne-Viru; PÕ—Põlva; RA—Rapla; TA—Tartu; VA—Valga; VI—Viljandi; VÕ—Võru. ** NA—not available.

**Table 2:** *CYP51* mutation frequencies (%) in *R. collo-cygni* population from Estonia in 2019 and 2020. Frequencies between 1‒20% are indicated in green, 21‒50% are yellow, 51‒100% are pink, missing mutations (0%) are grey.

County *	*CYP51* Mutation Frequency (%)								
	I381T		I384T		S459C		S459Y	S459T	S459L
	2019	2020	2019	2020	2019	2020	2020	2019	2019
IV	NA **	100	NA	100	NA	100	0	NA	NA
JÕ	97	78	97	78	89	46	54	0	0
JÄ	NA	100	NA	100	NA	37	63	NA	NA
LV	100	100	100	100	100	100	0	0	0
PÕ	NA	100	NA	100	NA	86	0	NA	NA
RA	NA	100	NA	100	NA	50	17	NA	NA
TA	100	70	100	70	89	100	0	11	0
VA	100	NA	100	NA	40	NA	NA	60	0
VI	NA	90	NA	90	NA	88	12	NA	NA
VÕ	83	100	83	100	83	100	0	0	17
Average	96	93	96	93	80	79	16	14	3

* Counties presented are: IV—Ida-Viru; JÕ—Jõgeva; JÄ—Järva; LV—Lääne-Viru; PÕ—Põlva; RA—Rapla; TA—Tartu; VA—Valga; VI—Viljandi; VÕ—Võru. ** NA—not available.

**Table 3:** Mutation frequencies (%) in *SdhC* gene in *R. collo-cygni* population from Estonia in 2019 and 2020. Frequencies between 1‒20% are indicated in green, 21‒50% are yellow, 51‒100% are pink, missing mutations (0%) are grey.

County *	*SdhC* Mutation Frequency (%)							
	H146R		N164H		G167C		V184L	
	2019	2020	2019	2020	2019	2020	2019	2020
IV	NA **	88	NA	13	NA	13	NA	50
JÕ	43	50	0	10	0	10	11	20
JÄ	NA	42	NA	21	NA	21	NA	21
LV	88	0	0	100	0	100	0	0
PÕ	NA	100	NA	0	0	0	NA	0
RA	NA	67	NA	50	NA	50	NA	50
TA	85	36	0	7	0	7	0	0
VA	40	NA	0	NA	0	NA	0	NA
VI	NA	57	NA	50	NA	50	NA	50
VÕ	58	58	0	0	0	10	0	0
Average	63	55	0	28	0	29	2	21

* Counties presented are: IV—Ida-Viru; JÕ—Jõgeva; JÄ—Järva; LV—Lääne-Viru; PÕ—Põlva; RA—Rapla; TA—Tartu; VA—Valga; VI—Viljandi; VÕ—Võru. ** NA—not available.

## Data Availability

Data are available upon request.

## Supplementary Materials

The following are available online at https://www.mdpi.com/article/10.3390/microorganisms9071514/s1, Table S1: PCR primers used for fungicide target gene amplification.
