# Supplementary material for: Fungicide Resistance Evolving in Ramularia collo-cygni Population in Estonia
Source: Microorganisms. 2021 Jul 15;9(7):1514. doi: 10.3390/microorganisms9071514 (PMC8307248; doi:10.3390/microorganisms9071514)
Supplement: Supplementary file 1 [file microorganisms-09-01514-s001.zip › microorganisms-1284927-supplementary.pdf]

Table S1. PCR primers used for fungicide target gene amplification.

Target gene	Primer name	Primer sequence (5´-3´)	Reference
Cytb	RCCcytobF	GGATGATTAATACGTTACTTACACTC	[30]
	RCCcytobR	GTTACCTGATCCTGCGCTGT	
SdhB	SdhB_Rcc_Final_F	CAAATCACACACCATCCAGT	[34]
	SdhB_Rcc_Final_R	CCAGCCCTCTTTACATCCTC	
SdhC	SdhC_Rcc_Final_F	CACTCCAGCAAACCACGACC	[34]
	SdhC_Rcc_Final_R	TAAAGCAGTTCTGTTGCTCT	
CYP51	KES2230	CCTCCCTCCAACCATCCATTCC	[33]
	KES2231	ATCGCGGAGTGCATAAAAT	
